# The Impact of Sea Ice Loss on Benthic Communities of the Makarov Strait (Northeastern Barents Sea)

**DOI:** 10.3390/ani13142320

**Published:** 2023-07-15

**Authors:** Lyudmila V. Pavlova, Alexander G. Dvoretsky, Alexander A. Frolov, Olga L. Zimina, Olga Yu. Evseeva, Dinara R. Dikaeva, Zinaida Yu. Rumyantseva, Ninel N. Panteleeva

**Affiliations:** Murmansk Marine Biological Institute of the Russian Academy of Sciences (MMBI RAS), 183010 Murmansk, Russia

**Keywords:** macrozoobenthos, environmental factors, sea ice extent, Barents Sea, Makarov Strait, St. Anna Trough

## Abstract

**Simple Summary:**

The warming of Arctic regions has led to an increase in extended ice-free periods in the continental shelf of the northeastern Barents Sea. A study on the Makarov Strait in the southwestern part of the St. Anna Trough in autumn 2019 investigated the effect of this process on the structure and functioning of benthic communities. It was found that the biodiversity and structure of local macrozoobenthos were connected with the duration of ice-free periods because this variable influenced vertical carbon flux and was found to be the primary predictor for faunal abundance and diversity indices. Two faunal groups were identified, corresponding to short and long open-water periods. Our results may have important implications for the conservation and monitoring of this region.

**Abstract:**

The continental shelf of the northeastern Barents Sea is presently experiencing a weak influx of Atlantic water from the west. In recent times, warming in Arctic regions has led to an increase in extended ice-free periods in this area, instead of significantly elevating water temperatures. The implications of this phenomenon on the structure and functioning of benthic communities were investigated during the autumn of 2019 within the Makarov Strait, located in the southwestern part of the St. Anna Trough. The macrozoobenthic communities exhibited a clear connection with the duration of ice-free periods. This variable influenced a vertical carbon flux, which subsequently served as the primary predictor for faunal abundance and diversity, as demonstrated by redundancy and correlation analyses. Two faunal groups were identified, corresponding to short and long open-water periods. Both groups had similar alpha diversity (65 ± 6 and 61 ± 9 species per station) and biomasses (39 ± 13 and 47 ± 13 g m^−2^) but displayed differing abundances (1140 ± 100 vs. 4070 ± 790 ind. m^−2^) and other diversity indices. We observed a decline in the proportion of polychaetes, accompanied by an increase in the proportion and diversity of bivalves, as well as a rise in the abundance of infaunal species, sub-surface deposit feeders, and mobile suspension feeders, in response to the increasing vertical carbon flux. The potential increase in anthropogenic pressures related to oil development in the northeastern Barents Sea highlights the importance of our study for conservation and monitoring efforts in the region.

## 1. Introduction

The Arctic Ocean encompasses 31% of the global ocean’s shelves, with 53% of these having a depth of less than 200 m [1]. This region is characterized by seasonal or permanent ice coverage, low temperatures, and pronounced seasonality, such as variations in light availability during the polar night and midnight sun, as well as fluctuating levels of organic material input. In this high-latitude environment, the survival of polar benthic fauna is greatly influenced by seasonality and the availability of food resources [1].

The Barents Sea is regarded as one of the largest continental shelves globally. The northern region of this sea is categorized by a cold Arctic climate, relatively low productivity, and ice-associated ecosystems, while the southern part encompasses warmer areas with highly productive ecosystems [2,3,4]. Despite the challenging environmental conditions here, marine sediments at varying depths host an array of diverse and abundant communities of highly adapted benthic fauna [5,6,7]. These benthic organisms are closely associated with large-scale water column processes that dictate the availability of food resources reaching the underlying sediments [8]. As the community patterns of these organisms are directly influenced by the exportation of organic matter from the overlying water column, benthic communities serve as long-term integrators of water column processes that can reflect different hydrographic regimes [9,10,11,12].

The role of benthos in the Barents Sea ecosystem is essential as seabed macrofauna is crucial in the overall production, turnover rates, and carbon remineralization of the system [4]. Benthic organisms participate in biochemical processes such as decomposition of organic matter near the sediment–water interface and sediment mixing, which influences the whole ecosystem. On the Barents Sea shelves, macrofauna communities frequently exhibit high diversity and achieve high biomass and production [5,9]. Many benthic species are prey for top predators including commercially important fish and shellfish species [13,14]. The Barents Sea’s high productivity supports extensive fisheries of Atlantic cod, capelin, haddock, beaked redfish, golden redfish, Greenland halibut, red king crab, snow crab, and northern shrimp [15,16,17], and contributes to the good aquaculture potential of the coastal zone [18,19].

Over recent decades, the seasonal Arctic sea ice cover has diminished at historically unprecedented rates [20]. This reduction in the sea ice cover is accompanied by a decrease in the sea ice thickness and a transition from multiyear to predominantly seasonal ice cover [21]. Changes in the seasonal sea ice conditions and seawater temperatures have been shown to significantly affect primary production regimes at high latitudes [22] and, consequently, pelagic–benthic coupling processes such as energy transfer from pelagic zones to benthic standing stocks [23].

In recent years, there has been a notable warming of the Atlantic water inflow, along with a concurrent retreat of the ice cover, resulting in an increased influence of Atlantic water on the Barents Sea. This phenomenon has been referred to as “Atlantification” [24]. Studies revealed that this process leads to greater growth rates and biomasses of benthic organisms, as well as range expansions of certain taxa, as evidenced through benthic surveys conducted in the southern, central, western, and eastern parts of the Barents Sea [5,25,26,27,28,29]. However, the northern Barents Sea and the continental slope that lies between the Novaya Zemlya and Franz Josef Land archipelagos (Makarov Strait), including the southwestern region of the St. Anna Trough, remain relatively understudied. The primary quantitative benthic data for these specific areas were collected during the years 1931–1932 [30] and 1994–1995 [31,32,33]. Based on the observations of these researchers, the local benthic fauna is characterized by a low biomass, primarily composed of echinoderms, which reflect an oligotrophic environment and the predominance of oxidized brown silt.

The Makarov Strait is characterized by a dynamic ecological environment that significantly influences the composition and abundance of benthic organisms, as well as the primary production regime. This region is situated within the continental slope, featuring a complex topography. Consequently, transformed Barents Sea water circulates into the Polar Basin by following the local troughs [34]. Notably, the vertical stratification and interaction among Arctic water, Barents Sea water, and the warmer, deeper Atlantic water remain prominently expressed. The Atlantic water enters the study area from the Arctic basin, coursing along the St. Anna Trough at a depth of 100–200 m and following the western slope [35,36]. Comprising fine-grained components such as silt and clay [37], the benthic sediments here possess a low content of organic matter [38,39]. The harsh environmental factors of the region, which include long polar nights, extended ice cover periods, short vegetation seasons of phytoplankton, low near-bottom oxygen content, and organic matter flux [40,41], contribute to the scarcity of food resources for local seabed communities. Access to the region is challenging throughout the year, particularly during cooling periods in the Arctic, when harsh ice conditions make the area inaccessible [42,43]. However, the recent warming period has led to a significant reduction in the ice cover [44], allowing for more straightforward access to the study area, and enabling researchers to evaluate the response of high-latitude bottom fauna to shifts in the ice regime and increased primary production [45,46].

Our study aims to describe spatial patterns in the diversity and abundance of benthic fauna in the northeastern Barents Sea and evaluate the role of environmental factors in determining the structure of local benthic communities under modern conditions of sea ice loss.

## 2. Materials and Methods

### 2.1. Sample Collection and Analysis

**Figure 1:**
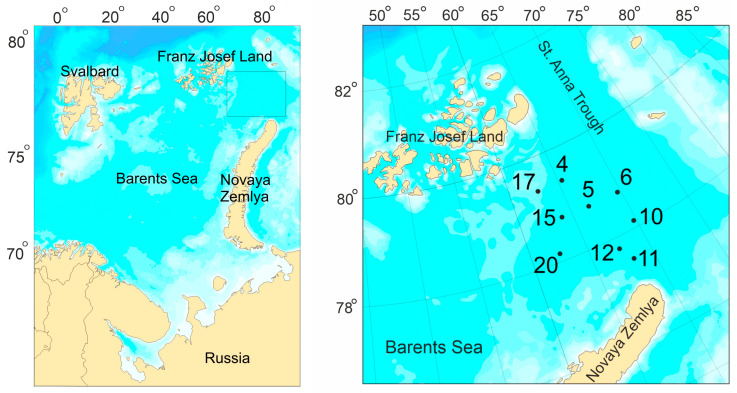
Location of sampling stations (with their numbers) in the northeastern Barents Sea, autumn 2019.

Macrozoobenthos sampling was carried out at 9 stations located on the western slope and bed of the St. Anna Trough and its southwestern branch, the Northeast Trough (Figure 1), during a research cruise of the R/V *Dalnie Zelentsy* between 30 October and 7 November 2019. At each site, three replicate samples were collected aboard the research vessel at depths of 186–507 m using a Van Veen grab (0.1 m^2^ sampling area).

The collected samples were washed through a 0.5 mm sieve and fixed with 4% neutral-buffered formalin. Sediment types were ascertained via visual examination according to color, consistency, and grain size using the criteria laid out by Istoshin [47] and Noorany [48]. Moreover, samples were thoroughly inspected for relative abundance, characterized as either low or high, of benthic foraminifera with agglutinated shells, with the underlying assumption that this particular attribute serves as an indirect indicator of the organic matter sedimentation level [49] and organic matter content. The latter parameter was assessed via differential preservation of foraminiferal assemblages and thickness of the oxidized layer in sediments, ranging from very weak to well expressed on a scale from 1 to 3. Vertical profiles of water temperature and salinity were recorded using a CTD Sealogger (SBE 19plus V2).

In the laboratory, the benthic samples were washed again, fixed in 75% ethanol, and identified to the lowest possible taxonomic level using the latest nomenclature from the World Register of Marine Species (http://marinespecies.org, accessed on 25 May 2023). Benthic organisms were counted and weighed, with a precision of 0.0001 g for wet weight. Mollusks were weighed with their shells, polychaetes that exhibited tube secretion were weighed with their tubes, and tube-dwelling encrusting polychaetes were weighed without tubes. For each station, the measured abundance (ind. m^−2^) and biomass (g m^−2^) values of three samples were averaged.

### 2.2. Diversity Indices

Several diversity indices were calculated, including frequency of occurrence of benthic taxa (FO), species richness (SR, mean number of species per sample), alpha diversity at each station (number of taxa per 0.3 m^−2^), Shannon index (H’), Pielou evenness (J’), Simpson index (D’), difference of evenness index (*D_E_*) [26], the expected number of species among 100 individuals (ES_100_) [50], and the total expected number of species (Chao2 index).

The *D_E_* index, an indicator of the ecological status at a station, was calculated as follows:

$$D_{E}=\frac{J_{A}-J_{B}}{\mathrm{lg}S}$$

where *J_A_* and *J_B_* are Shannon diversity indices calculated by abundance and biomass, respectively, and *S* is the total number of species in a sample. *D_E_* ranges from −1 (no stress) to +1 (very strong stress), and 0 is the transition point from the unstressed to the stressed state.

To assess the contributions of each taxon to the energy flow of the community, its respiration (metabolic) rate was calculated using the following formula proposed by Golikov et al. [51]:

$$R=cN^{0.25}\cdot B^{0.75}$$

where *N* is the abundance (ind. m^−2^), *B* is the biomass of the taxon (kJ m^−2^), and *c* is the specific coefficient showing the intensity of metabolism (J h^−2^). We used the following coefficients for particular groups: Hydrozoa (0.59), Actinaria (0.42), Polychaeta Errantia (3.10), Polychaeta Sedentaria (2.10), Amphipoda (2.89), Isopoda (2.89), Ostracoda (1.51), Gastropoda (1.76), Bivalvia (2.10), Astartidae (0.84), Mytilidae (3.35), Tellinidae (1.38), Echinodermata (0.67), Bryozoa (0.59), Tunicata (0.42) [51].

### 2.3. Statistical Analysis

To visually depict the distribution of sampling stations in relation to specific environmental conditions, a non-metric multidimensional scaling method based on the Euclidean distance similarity matrix was employed. These conditions included depth, temperature, salinity, sediment type, vertical organic matter fluxes, and the duration of the ice-free period. The latter was calculated using data obtained from the Arctic and Antarctic Research Institute’s ice coverage archive (http://old.aari.ru accessed on 25 May 2023) for the five years preceding the study period. Prior to the analysis, all environmental variables were log(x + 2) transformed to reduce skewness and to homogenize variances.

For the purpose of distinguishing spatial communities, cluster analysis was conducted utilizing the Bray–Curtis similarity matrix of benthic respiration rates, with group average linkage classification. Prior to analysis, data were log(x + 1) transformed, and rare or large species with random distributions were excluded from the analysis. Similarities between station groups, based on hierarchical clustering, were tested using analysis of similarities (ANOSIM). A score of 0 for global R signifies no separation, whereas a score of 1 indicates a complete separation of groups [52]. To identify species responsible for differences between station groups, SIMPER analysis was employed [52]. Benthic communities/associations were categorized according to dominating taxa (R > 10%), with species forming the core of the community regarded as characteristic species. To ascertain the trophic structure of the communities, biomass values of sessile or semi-mobile taxa with uniform distributions were evaluated, while large mobile invertebrates with random distribution patterns were excluded from analysis. Classification of benthic species’ life history traits was obtained from the existing literature [53,54,55]. To assess differences in abundance, biomass, diversity indices, and mean weights of specific taxa between station groups, non-parametric Mann–Whitney U-tests were carried out. All calculations were performed using the software package PAST 4.12 [56].

To examine the relationships between local environmental variables and benthic abundances, biomasses, and diversity indices, we conducted a Redundancy Analysis (RDA). This approach was selected based on preliminary detrended correspondence analysis, which indicated that the length of the first axis was <3 standard deviation units. This signified that the linear ordination method was preferable over the alternative methods [57]. The environmental dataset used in the analyses contained the aforementioned parameters, while three distinct datasets were implemented to quantify response variables. Two datasets included abundances and biomass of the most common species, while the third dataset included diversity indices. A Monte Carlo permutation test (*n* = 999) was conducted to elucidate the explanatory variables that best explained the benthic abundance, biomass, and diversity data. CANOCO for Windows v. 4.5 was utilized for all ordinations [57]. Prior to conducting the analyses, the species abundance and biomass datasets were log (x + 1) transformed to minimize the effect of high values on the analyses. In addition, non-parametric Spearman rank correlations were calculated to assess the relationships between selected environmental and biological variables.

Mean values are presented with standard errors.

## 3. Results

### 3.1. Environmental Conditions

During the study period, two distinct water masses were observed. The first, transformed Barents Sea water (BSW), is primarily composed of Atlantic water (AW) which has undergone heat loss. This water mass was detected at eight sampling stations (Stations 4, 5, 6, 10, 11, 12, 15, and 20) characterized by negative water temperatures and a salinity of 34.9 psu. The second water mass, Arctic water (ArW), was observed at Station 17, with lower temperature and salinity levels compared to the BSW. Station 4 had a lower salinity level than the other stations belonging to the BSW group, possibly indicating a transition zone between the BSW and ArW. The bottom sediments were mainly composed of oxidized brown silt or sandy silt (Table 1).

Variations in the thickness of the oxidized layer, as well as disparities in the postmortem status of foraminiferal assemblages, suggest a heterogeneous influx of organic matter from upper water layers to the seafloor. Weaker organic matter fluxes were observed at Stations 4, 5, 6, 15, and 17, where foraminiferal sand and thick layers of brown silt were found overlaying a lower layer of brown clay. In comparison, more robust sedimentation processes occurred at Stations 10, 11, 12, and 20. These stations exhibited a thin layer of sediment overlaying gray sediments, and a poor postmortem preservation of foraminifera was noted. This indicated a positive redox potential, which facilitated shell degradation in these organisms. Stations 11 and 12, in particular, had the thinnest layers of oxidized silt. Additionally, at Stations 10, 11, 12, and 20, the duration of ice-free periods was twice as long compared to the remaining stations (Table 1).

**Figure 2:**
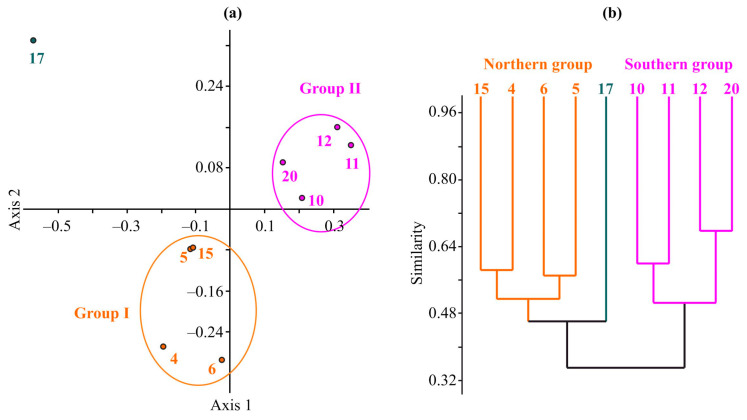
Dendrograms resulting from nMDS ordination based on the Euclidean distance matrix of environmental conditions (**a**) and from clustering performed on the Bray–Curtis similarity matrix produced from log(x + 1) transformed metabolism rate data (**b**).

The nMDS revealed a distinct separation between Station 17, situated within the ArW region, and the remaining stations, which were located in the BSW area (Figure 2a).

The BSW stations were further divided along Axis 2 into two distinct groups. The first group, Group I, encompassed Stations 4, 5, 6, and 15, and was characterized by a short ice-free duration (ranging from 3 to 4 months) and a weak vertical influx of organic matter. Conversely, Group II consisted of stations exhibiting prolonged ice-free periods and higher sedimentation levels. The dissimilarities between the two station groups were found to be statistically significant according to the ANOSIM test (R = 0.993, *p* = 0.0027). Furthermore, the SIMPRER test indicated that the primary factors contributing to the dissimilarity between Groups I and II included sediment type (accounting for 30.5% of the difference), ice-free period duration (29.0%), and vertical influx of organic matter (26.6%). In contrast, water temperature and depth served as the predominant factors driving the dissimilarity between Group I and Station 17, contributing 42.2% and 41.2%, respectively. Additionally, these factors were responsible for the dissimilarity between Group II and Station 17, with contributions of 27.0% and 29.0%, respectively.

### 3.2. Benthic Diversity, Abundance, and Biomass

A total of 201 benthic taxa from 12 phyla were identified amongst the 27 samples that were collected across our sampling stations (Appendix A). The Chao2 index for the study area yielded a result of 256. The most diverse groups were Polychaeta and Crustacea, with 72 and 43 species accounting for their respective levels of SR. The mean SR was found to be 37 per sample, while the alpha diversity averaged 64 species per station. The highest SR was observed at Station 5, while the minimum was identified at Station 20 (Table 2). The mean benthic abundance and biomass were calculated to be 2450 ± 610 ind m^−2^ and 42 ± 9 g m^−2^, respectively. It is worth noting that the abundance had a tendency to vary to a lesser degree than the biomass, with coefficients of variation ranging between 0.01–0.54 and 0.15–1.32, respectively.

Regarding the frequency of occurrence (FO), there were a total of 20 species that had a dominating presence (FO greater than 50%), which included *Prionospio cirrifera* and *Abyssoninoe* cf. *abyssorum* within the Polychaeta group, the cumacean *Ektonodiastylis nimia*, and the bivalves *Yoldiella nana* and *Mendicula ferruginosa* (Table 3).

Meanwhile, the number of sub-dominant species (with FO between 25% and 50%) was 30, whereas the remaining 150 species had rare occurrences (FO less than 25%). Species that were classified as dominating in terms of their occurrence provided 37% of the total biomass and 71% of the total abundance, while the sub-dominant species contributed 11% and 18%, respectively, and rare groups contributed 52% to the total biomass and 11% to the total abundance.

### 3.3. Faunal Groups

**Figure 3:**
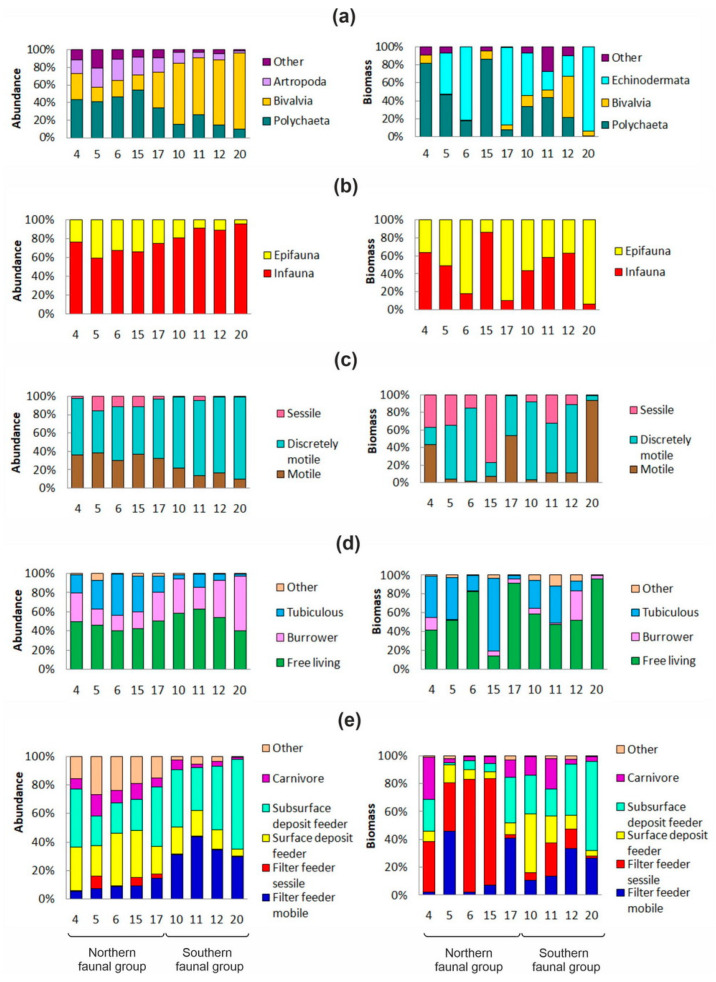
Structure of benthic communities represented as contributions of the main benthic groups to the total material in the Makarov Strait: (**a**) taxonomic structure, (**b**) bottom surface position, (**c**) mobility, (**d**) life history traits, (**e**) trophic structure. Left side—benthic abundance, right side—benthic biomass.

Spatial variations in the diversity, abundance, and biomass of the benthic taxa were prominently observed throughout the study area, as illustrated in Figure 3.

A cluster analysis of the benthic metabolic rates reflected the outcomes of the nMDS based on environmental variables, revealing two distinct station groups, northern (=Group I) and southern (=Group II), which exhibited a 36% similarity level (Figure 2b). These groups were characterized by differences in the abundance of dominant species, such as *Parathyasira equalis*, *Spiochaetopterus typicus*, *Mendicula ferruginosa*, *Yoldiella nana*, and *Aglaophamus malmgreni*, as well as differences in the abundance of sub-dominant and rare species. Specifically, the sipunculan *Golfingia (Golfingia) vulgaris vulgaris*, the polychaetes *Cistenides hyperborea*, *Leitoscoloplos acutus*, and *Levinsenia gracilis*, the cumacean *Eudorella emarginata*, and the bivalves *Nuculana pernula*, *Ennucula tenuis*, *Macoma calcarea*, and *Yoldiella frigida* were uniquely identified at stations associated with the southern complex. In contrast, the polychaete *Notomastus latericeus*, the bivalve *Lyonsiella abyssicola*, and the cumacean *Eudorella gracilis* were solely documented at stations pertaining to the northern complex.

No significant differences were detected between the northern and southern groups regarding the species richness (SR) (38 ± 4 vs. 37 ± 6), alpha diversity (65 ± 6 vs. 61 ± 9), and total biomasses (39 ± 13 and 47 ± 13 g m^−2^) according to the Mann–Whitney test (*p* > 0.05). However, the average abundance was observed to be lower at stations from the northern part of the study area (1140 ± 103 vs. 4070 ± 788 ind. m^−2^) (*p* < 0.05). Furthermore, polychaetes primarily dominated in the northern region, while bivalve mollusks were predominantly observed in the southern region (Figure 3a). In the latter group, *Yoldiella nana* and *Mendicula ferruginosa* emerged as the most prevalent components, with abundances reaching 1490 and 1057 ind. m^−2^, respectively (at Station 10). In comparison, the northern station group exhibited higher abundances of epifaunal taxa, mobile taxa, tubiculous taxa, and surface deposit feeders. In contrast, the southern group was distinguished by a greater presence of semi-mobile infaunal organisms, including free-living and burrow-dwelling taxa, as well as mobile suspension feeders and sub-surface deposit feeders (Figure 3c–e).

The contributors with the most significant impact on the total biomass were polychaetes and echinoderms. In the northern complex, polychaetes comprised 47% of the total biomass, while echinoderms made up 42% of the total biomass. In contrast, the southern complex was dominated by echinoderms with 46%, followed by polychaetes (25%) and bivalves (18%). Furthermore, it was observed that sessile suspension feeders had higher biomasses at the northern stations, while the southern stations were dominated by surface and sub-surface deposit feeders. Benthic abundance was found to be non-randomly distributed, particularly in the southern complex. This phenomenon was characterized by low H’ (3.4 ± 0.2), J’ (0.58 ± 0.02), and ES_100_ (0.18 ± 0.09), and a relatively high D’ index (0.19 ± 0.02). Intermediate and slightly negative values for the *D_E_* index were recorded at Stations 10, 11, and 12, respectively, indicating a shift towards imbalance communities. Conversely, the northern station group displayed more diverse benthic communities without any signs of damaged structure, with higher values of H’ = 4.90 ± 0.16, J’ = 0.81 ± 0.02, ES_100_ = 0.43 ± 0.19, and a lower value of D’ = 0.06 ± 0.01. A further analysis revealed significant differences between the two station groups in terms of the total benthic abundance, abundance and biomass of bivalves, abundance of infauna, abundance and biomass of subsurface deposit feeders, and abundance of mobile suspension feeders, as well as H’, J’, D’, and ES_100_ (Mann–Whitney U-tests, *p* < 0.02 in all cases).

### 3.4. Benthic Communities within the Faunal Groups

The ANOSIM test indicated that there were sub-groups of stations found in both the northern and southern faunal complexes, and significant differences were observed among them (*R* = 0.984, *p* = 0.0013). The benthic communities dominated by the polychaete *Spiochaetopterus typicus* were found to occur over a wide area (Table 4). Sub-groups of these communities were observed at stations with long ice-free periods and were occupied by the *Spiochaetopterus typicus* + *Laonice cirrata* community and the *Yoldiella nana* community, where *Maldane sarsi* (9.2%), *Laonice cirrata* (8.9%), *Yoldiella lenticula* (7.9%), and *Spiochaetopterus typicus* (7.3%) were the most important contributors to dissimilarity between these communities.

At stations with shorter ice-free periods, two other communities, *S. typicus* + *Ophiopleura borealis* and *S. typicus* + *Aglaophamus malmgreni* + *Abyssoninoe* cf. *abyssorum*, were found to exist, and they are characterized by a high diversity and low abundance. The communities differed mainly due to different contributions of dominant species, such as *A. malmgreni* (12.2%), *A.* cf. *abyssorum* (8.7%), *Spiophanes kroeyeri* (4.1%), and *Yoldiella lenticula* (3.5%), as well as some rare species. Although Station 17 was a variant of the *S. typicus* community typical of the northern complex, it did not group with the other northern sub-groups at a similarity level of 50%. The community of *S. typicus* + *Laonice cirrata* located in the southern part of the St. Anna Trough had the highest faunal abundance (Table 4). Moderate alterations in the community structure were observed due to the high abundance of certain taxa, which was reflected in a polydominance pattern in the trophic structure, low H’ and J’, high D’, and intermediate *D_E_* values. Meanwhile, other *S. typicus* communities displayed normal diversity indices. On the other hand, the *S. typicus* + *Aglaophamus malmgreni* + *Abyssoninoe* cf. *abyssorum* community on the western slope of the St. Anna Trough had low faunal abundances and biomasses, as well as a low proportion of epifauna (Table 4).

The community of *Spiochaetopterus typicus* + *Ophiopleura borealis* had a high SR and biomass but a low abundance, while the community of *Yoldiella nana* had the lowest SR but harbored the maximum number (17) of bivalve species. Interestingly, even though the sea urchin *Strongylocentrotus pallidus* had a high contribution to the total biomass and rate of metabolism in this community, it was excluded from the community’s name as it was only found in one station (Station 20). Although there was low diversity, alterations in the community structure were less expressed (Table 4). Furthermore, the area with a longer ice-free period was dominated by sub-surface deposit feeders and surface deposit feeders, while sessile suspension feeders became the dominant group in the northern area with a shorter ice-free period, with a surprisingly high contribution to the total material. Finally, mobile suspension feeders were the most common at Station 17.

### 3.5. Environmental Factors driving Benthic Communities

**Figure 4:**
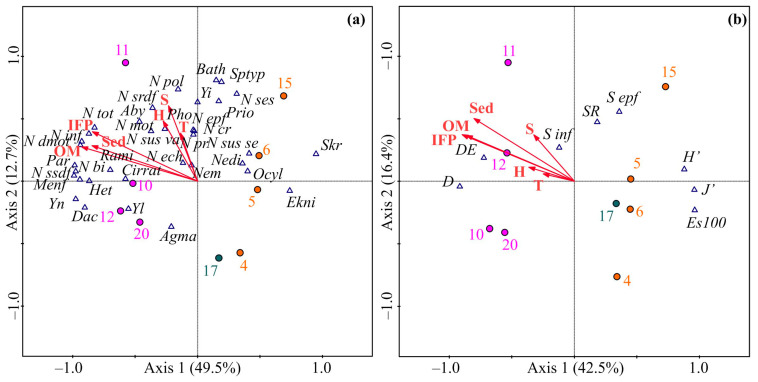
Ordination of samples by redundancy analysis with respect to benthic abundance (**a**) and diversity (**b**) and their relations to environmental variables in the northeastern Barents Sea. The proportions of the total variability explained by the first two axes are given. Biological variables: Ekni—*Ektonodiastylis nimia*, Rami—*Rabilimis mirabilis*, Nedi—*Nephasoma (N.) diaphanes diaphanes*, Nem—Nemertini g. sp., Aby—*Abyssoninoe* cf. *abyssorum*, Agma—*Aglaophamus malmgreni*, Cirrat—Cirratulidae g. sp., Het—*Heteromastus filiformis*, Ocyl—*Ophelina cylindricaudata*, Pho—*Pholoe assimilis*, Prio—*Prionospio cirrifera*, Styp—*Spiochaetopterus typicus*, Skr—*Spiophanes kroeyeri*, Bath—*Bathyarca glacialis*, Dac—*Dacrydium vitreum*, Menf—*Mendicula ferruginosa*, Par—*Parathyasira equalis*, Yi—*Yoldiella intermedia*, Yl—*Yoldiella lenticula*, Yn—*Yoldiella nana*, SR—total number of species, H’—Shannon index, J’—Pielou index, D—Simpson index, ES100—expected number of species among 100 individuals, S inf—number of infaunal species, S epf—number of epifaunal species, Mob—mobile, N—abundance, B—biomass, inf—infauna, epi—epifauna, mot—mobile, dmot—semi-mobile, ses—sessile organisms, sus ses—sessile suspension feeders, sus vag—mobile suspension feeders, srdf—surface deposit feeders, ssdf—sub-surface deposit feeders, pr—omnivores, bi—Bivalvia, cr—Crustacea, ech—Echinodermata, pol—Polychaeta. Environmental variables: H—depth, T—temperature, S—salinity, Sed—sediments, OM—organic matter influx, IFP—duration of ice-free period.

The RDA model, conducted on the basis of the abundance data of the benthic fauna, yielded statistically significant results (verified by the test of significance of all canonical axes as follows: trace = 0.759, F-ratio = 5.782, *p* = 0.003), where the first and second axes accounted for 62.2% of the total variance. Axis 1 exhibited strong negative correlations with all environmental variables (up to 0.9), while Axis 2 had positive associations (up to 0.6) with these variables (Figure 4a).

The first axis separated the stations of the southern complex, which showed an environment richer in trophic resources, from those of the northern complex, which exhibited oligotrophic conditions. The second axis segregated the sampling sites as per their depth, with deeper stations found in the upper side of the ordination plot, and shallower ones positioned in the lower side.

In contrast to the former, the RDA model based on the biomass data revealed insignificant relationships between the explanatory and biological variables (test of significance of all canonical axes as follows: trace = 0.741, F-ratio = 0.956, *p* = 0.550) (ordination not shown).

The RDA model based on the diversity data generated a significant model (test of significance of all canonical axes as follows: trace = 0.617, F-ratio = 2.953, *p* = 0.045), where the first two axes explained 58.9% of the total variation (Figure 4b). Axis 1 differentiated the sampling stations according to the durations of ice-free periods and their associated parameters, with the southern cluster having negative RDA 1 scores, and the northern cluster having positive RDA 1 scores. Axis 2 separated the stations based on their depth.

The forward selection procedure showed that trophic conditions, particularly the vertical flux of organic matter, were the primary factor contributing to the observed variations in both the abundance and diversity data (Table 5). Specifically, a higher influx of organic matter resulted in an increase in the abundance of benthic taxa; however, it reduced SR, H’, and J’, and led to more stressed benthic communities with a stronger dominance of some major taxa, resulting in higher D’ and *D_E_* indices.

The results from the correlation analysis are consistent with those from the RDA, confirming the observed patterns in the data. The bivalve species, including *Parathyasira equalis*, *Y. nana*, *Mendicula ferruginosa*, and *Dacrydium vitreum*, as well as the polychaete *Heteromastus filiformis* and the ostracod *Rabilimis mirabilis*, exhibited a positive association with vertical organic matter flux, in addition to its associated parameters, namely, the substrate type and the duration of the ice-free period (*p* < 0.040 in all cases). Conversely, the polychaete worm *Spiophanes kroeyeri* and the cumacean *Ektonodiastylis nimia* demonstrated a negative correlation with these factors, thus indicating a preference for more oligotrophic conditions. The water temperature appeared to have a positive influence on the abundance of the polychaete *Pholoe assimilis* (*p* = 0.032); however, a negative relationship was observed between this factor and the abundance of the polychaete *Ophelina cylindricaudata* (*p* = 0.030).

In terms of biomass, our analysis showed that the bivalves exhibited positive relationships with all three factors related to trophic conditions (*p* < 0.007). This pattern was also evident for the *R. mirabilis*, *Y. nana*, *M. ferruginosa*, and *P. equalis* polychaetes belonging to the family Cirratulidae, and *H. filiformis*, with *p* < 0.02 in all cases. Furthermore, the biomass of the sub-surface deposit feeders was found to be higher at the stations exhibiting greater values of vertical organic matter flux (*p* = 0.009) and those containing sediments with a thin layer of oxidized silt (*p* = 0.016). Similar to the patterns observed for abundance, the biomasses of *E. nimia* and *S. kroeyeri* were negatively correlated with trophic conditions (*p* < 0.02). Lastly, our results indicate that higher water temperatures led to higher biomasses of *Dacrydium vitreum* (*p* = 0.039), yet resulted in lower biomasses of *Ophelina cylindricaudata* (*p* = 0.013).

## 4. Discussion

### 4.1. Environment in the Makarov Strait under Warming Conditions

The Arctic region is undergoing considerable warming, and since the 2000s, there has been a remarkable decrease in the sea ice extent and ice cover durations across most Arctic seas. In particular, the Barents Sea has witnessed a significant decline in the ice cover duration [58,59], with the highest rate of reduction (65 days per decade) observed in the northeastern part of the region, including the Makarov Strait [44].

The study area displays considerable heterogeneity in terms of the timing and pattern of ice clearance, as well as the duration of the open water season. Currently, there are two distinct zones with different ice-free period durations; one is located south of the 78°30’ N conventional boundary, which exhibits a lengthy ice-free season of 7.3 months, and the other is situated north of this boundary, where the ice-free period extends for 3.8 months. These values exceed those recorded during the 1980s under cooling Arctic conditions when ice-free seasons persisted for less than two months in the southern locations and for one month in the northern locations, respectively [42].

The quality and quantity of primary production, including ice algae and phytoplankton that reach the seafloor, exert a profound influence on benthic communities [60]. This impact further cascades throughout the entire food web. It is well established that a permanent solid ice cover leads to a reduction in the vertical circulation of water masses and a consequent decline in primary production due to the impaired capacity of phytoplankton to thrive under unfavorable conditions. This results in a lower latitudinal migration of organic matter. As a consequence of these diminished carbon fluxes, the seabed habitat conditions become oligotrophic. A protracted ice-free season would facilitate an increased spatial availability for phytoplankton populations, an extended vegetation period, enhanced primary production, and a greater vertical carbon influx [61,62,63]. Furthermore, an extended ice-free period entails heightened wind-induced mixing of seawater, thereby promoting intensive pelagic–benthic processes associated with the export of organic matter from the surface layer to the seafloor and ensuing greater nutrient availability for benthic organisms [62,64].

The process of primary production, which drives the growth of marine organisms, is supported by additional carbon sources derived from frontal zones [65,66], water currents of Atlantic origin [63], glaciers, and meltwater, Pokrovsky et al., 2012 [61]. Climate forcing has also led to significant inputs of terrigenous organic matter into the seawater [61,67], thus enhancing primary production. However, the increased inflow of AW into the Barents Sea has had a limited impact on the thermal regime of the northeastern part of the area. An increase in water temperature, compared to normal and cold periods (1950–1998), was only observed in the upper 0–50 m layer [41], which is likely due to the displacement of warmer AW, whose influence on the system is significantly attenuated in the northeastern Barents Sea by cooled and saline ArW [68,69].

It is well established that the thickness of the oxidized layer in sediments reflects the intensity of the sedimentation processes, and a thick layer indicates low sedimentation rates of organic matter and fine particles [70]. A thick oxidized layer also provides favorable conditions for the preservation of agglutinated foraminiferal shells and foraminiferal sand, with a low content of live protists, which typically occurs in upper sediment layers [38]. These conditions were identified at stations belonging to the northern faunal complex. In contrast, a higher sedimentation rate of organic matter, along with the microbial decomposition of organic matter, promoting reduction processes, results in the formation of a reduced layer of gray sediments underneath a thin (less than 2 cm) layer composed of brown silt. Owing to bioturbation activity, dead foraminiferal shells fall into these sediments, where their destruction was observed [38]. The described benthic habitats with an intense vertical flux of organic matter occurred at stations belonging to the southern complex.

### 4.2. Functioning of Macrobenthic Communities under Modern Climatic Conditions

In general, the benthic fauna identified in the study area primarily consisted of species that are widely distributed across the Barents Sea shelf. Simultaneously, a minimal proportion of abyssal species, including the amphipods *Centromedon calcaratus* and *C. typhlops* and the cumacen *Leptostylis gorbunovi*, was also observed. These species are typically found in deeper regions but were identified at the St. Anna Trough due to their vertical migrations to depths of 400–500 m. Interestingly, the low abundance of the majority of species and the predominance of small-sized taxa accounted for over 50% of the rare species contributing to the total community biomass. This finding contrasts with other shelf areas of the Barents Sea, where dominant taxa typically provide 70–80% of biomass [71].

In terms of the mean abundance, the contemporary benthic fauna in the Makarov Strait closely resembles that of deep-water shelf areas of the Barents Sea [5,72], the northern section of the sea [73], and the western coast of Svalbard, which is occupied by ArW [25]. However, the average benthic biomass in our study area was found to be 1.5–2 times lower than in these aforementioned locations. Furthermore, a four-fold difference in the benthic biomass was observed when comparing our study area with more southern shelf locations [5,26] or the eastern part of the Barents Sea [74]. These notable spatial differences most likely reflect variations in the vertical carbon fluxes and nutrient concentrations available for benthic organisms across the Barents Sea.

According to our findings and previous research, the mean abundances of benthic taxa in the Makarov Strait in 2019 and the northern part of the shelf in 2016 [73] were twice as high as in the northern Barents Sea in 2003 [5]. This trend suggests that benthic organisms have exhibited positive responses to the enhanced primary production observed in the past 13–15 years due to the sea ice loss.

Our results reveal substantial variations in the composition, structure, and abundance of macrozoobenthos at stations with differing ice-free periods and vertical organic matter fluxes. These variations seem to be closely linked to differing trophic conditions, encompassing both nutrient quality and availability on the seafloor. Nutrients are widely considered primary drivers of the composition, structure, and functionality of benthic communities [75,76]. Moreover, direct associations between the productivity of pelagic communities and benthic abundances and biomasses were demonstrated for various Arctic shelf areas [23,25,26,77,78]. Additionally, increasing organic matter fluxes have been known to cause declines in diversity and evenness among infaunal benthic communities [60]. This pattern was observed in the southern faunal complex, most likely in response to a longer ice-free season and increased primary production associated with this development, as revealed by both the RDA and correlation analysis regarding the abundance, biomass, and diversity, particularly in cases of sub-surface deposit feeders and mobile suspension feeders. Our RDA models explained about 60% of the total variation in abundance and diversity patterns in the Makarov Strait. This means that other factors may have acted as drivers of the benthic community structure in this region. Although we cannot be certain of which additional factors caused the changes, spatial variability in abundance could be a result of biotic interactions such as competition for food and space, as well as predation. Other abiotic factors may also be responsible for these variations, including oxygen levels, levels of other essential elements, and water dynamics. At the same time, anthropogenic disturbance did not seem to be important because this area is not currently experiencing significant impacts from human activities with negligible pollution levels [79].

Currently, sub-surface deposit feeder communities within the southern faunal complex occupy a more expansive area when compared to the data from 1994 [31]. Generally, benthic communities of the continental slope are known to be dominated by suspension feeders [80], but our observations indicate a shift in the structure of seafloor communities. This shift reaffirms the increase in organic matter content both in the near-bottom environment (fresh nutrients) and in the sediments (semi-decayed organic matter) [81,82].

In our study, all the diversity indices employed were sensitive to the changes in the benthic community structure. In the area characterized by a lower productivity of pelagic communities, the indices of SR, H’, and ES_100_ were found to be higher, while D’ was lower compared to the regions with high primary production. This is because unfavorable nutrient conditions hinder a limited number of benthic taxa from reaching high abundances and displacing rare species. Similar patterns were observed in ice-free and seasonally ice-covered areas in the western and central Barents Sea [5,9,78]. However, in our study, the spatial differences between the two areas with varying ice conditions were much more pronounced compared to those observed at lower latitudes.

We found that the alpha diversity remained consistent within the study area, irrespective of the duration of the ice cover season. This outcome may be interpreted as an indication of the relatively low impact of recent warming on the faunal structure. Concurrently, although the lists of dominant species are similar, some discrepancies in sub-dominant compositions were already observed, allowing us to predict further changes in the macrozoobenthic structure in this area. Currently, the structure of the northern complex delineated in our research is quite consistent with that in the northern Barents Sea, where low primary production regions have benthic communities dominated by polychaetes. Moreover, we can observe a similarity between our southern complex and the southern part of the more productive Barents Sea shelf, where a prevalence of bivalve mollusks was detected [5].

Certain benthic communities that we identified in the Makarov Strait were previously described (with some modifications) in the adjacent Kara Sea shelf during the period of 1930–1940 [83]. Specifically, the authors documented a community consisting of *S. typicus* + *M. sarsi* + *A. crenata* at the northern tip of Novaya Zemlya, an *O. borealis* community along the southeastern slope of the St. Anna Trough, and an *O. borealis + Elpidia glacialis* community in the St. Anna Trough. In our samples, *S. typicus* were common but exhibited low abundances, *O. borealis* were less frequent than in the previous period, and the holothurian *E. glacialis* was not present. It is noteworthy that the bivalve mollusk *A. crenata* demonstrated a significantly lower occurrence in our samples, whereas it dominates by metabolism in the northern part of the Barents Sea shelf [74]. The low contribution of relatively large species such as *O. borealis* and *A. crenata* may be attributed to a shift in environmental conditions, which is associated with warming processes or the potential impact of the introduced snow crab, *Chionoecetes opilio*, whose juveniles were found at Station 11. The feeding activity of this predator is confirmed by the presence of cut pieces of body discs and partially eaten arms of the brittle star *O. borealis*, as well as shell fragments of large bivalves (*Bathyarca glacialis* or *E. tenuis*) found in our samples. Consequently, the productivity of the benthic communities might be underestimated in our study area, as we did not consider the benthic material consumed by snow crabs. It should also be noted that other potential bivalve predators such as deep-sea demersal fish have negligible abundances in the northern Barents Sea [84].

Given the possible increase in anthropogenic pressures associated with oil development in this region [85], our data may have important implications for further monitoring and conservation.

## 5. Conclusions

The process of Atlantification in the Barents Sea has led to a reduction in the duration of ice cover periods within the Makarov Strait. This particular factor has intensified production processes within pelagic communities, consequently increasing the vertical flux and instigating a shift in the functionality of benthic communities. In comparison, variables such as temperature, salinity, and depth did not exhibit a significant influence on benthic organisms. The sampling stations positioned south of the 78°30’ N latitude demonstrated higher faunal abundance and biomass levels, predominantly amongst sub-surface deposit feeders and mobile suspension feeders such as bivalves. Concurrently, alterations in the dominance structure were observed, indicating a transition towards a community status characterized by increased stress. These changes were associated with variations in the proportion of different trophic groups. Despite these developments, the study area remains oligotrophic when compared to the more productive regions of the Barents Sea shelf. Due to the unstable nature of habitat conditions, the local benthic fauna remains susceptible to various stress-inducing factors. Our research has shown that the structure of benthic communities in the Makarov Strait can serve as a valuable indicator of environmental conditions. The information obtained holds significant relevance for the ongoing monitoring of long-term ecosystem changes in the northern Barents Sea, influenced by factors such as climate change and anthropogenic activities associated with oil development. Considering the current trends in climate forcing, it can be anticipated that further modifications to the structure of benthic communities will occur in the high-latitude regions of the Barents Sea and adjacent areas of the Kara Sea.

## Figures and Tables

**Table 1:** Environmental conditions at sampling stations in the Makarov Strait, Barents Sea, autumn 2019.

No.	H	T, °C	S	Sediment		IFP	OM Influx
				Characteristics	Score		
4	427	−0.25	34.79	Liquid brown silt, foraminiferal sand, soft brown clay	1	3.0 ± 0.3	1
5	343	−0.2	34.9	Brown sandy silt, foraminiferal sand, soft brown clay	2	4.4 ± 0.5	1
6	507	−0.2	34.9	Brown silt, foraminiferal sand, soft brown clay	1	4.6 ± 0.6	1
10	445	−0.1	34.9	Brown sandy silt, gray clay	3	6.8 ± 1.1	2
11	502	−0.3	34.9	Thin layer of brown silt, gray clay, stones (occasional)	4	7.8 ± 1.2	3
12	430	−0.2	34.9	Thin layer of brown silt, gray clay, stones (occasional)	4	7.6 ± 1.2	3
15	361	−0.3	34.9	Brown sandy silt, foraminiferal sand, soft brown clay	2	4.2 ± 0.4	1
17	186	−1.2	34.7	Brown sandy silt, foraminiferal sand, brown clay	2	3.2 ± 0.6	1
20	392	−0.3	34.9	Brown sandy silt, gray clay, low content of Foraminifera	3	7.0 ± 0.9	2

Note: H—depth (m), T—temperature (°C), S—salinity, IFP—mean duration (±SE) of the ice-free period. Sediment scores represent values used in statistical analyses. Organic matter influx (OM) was assessed indirectly based on preservation of foraminiferal assemblages and thickness of the oxidized layer in sediments.

**Table 2:** Macrozoobenthic characteristics in the Makarov Strait, Barents Sea, autumn 2019.

No.	SR/α	ES_100_	H’	J’	D	*D_E_*	*N*	*B*	Main Contributors (%)		
									*N*	*B*	*R*
4	26/48	6.2	4.6	0.84	0.06	−0.23	770	5	*Yoldiella nana* (15%)	*Aglaophamus malmgreni* (25%) *S. typicus* (35%)	*A. malmgreni* (23%)
5	49/85	6.2	5.4	0.84	0.04	−0.46	1370	41	*Prionospio cirrifera* (8%) *Y. nana* (8%)	*S. typicus* (32%)	*S. typicus* (44%)
6	40/67	5.4	5.0	0.82	0.05	−0.66	1230	82	*P. cirrifera* (11%) *Spiochaetopterus typicus* (11%)	*Ophiopleura borealis* (81%)	*S. typicus* (48%)
10	43/68	1.5	3.6	0.58	0.17	−0.10	4640	39	*Y. nana* (32%) *Mendicula ferruginosa* (23%)	*O. borealis* (47%)	*Laonice cirrata* (15%), *Y.nana* (15%)
11	46/76	1.4	3.8	0.61	0.15	0.01	5580	72	*M. ferruginosa* (31%) *Y. nana* (18%)	*S. typicus* (20%)	*S. typicus* (25%)
12	41/69	1.6	3.5	0.58	0.19	0.06	4190	15	*Y. nana* (33%) *M. ferruginosa* (25%)	*O. borealis* (23%)	*Y. nana* (23%)
15	37/61	5.4	4.9	0.83	0.05	−0.51	1120	18	*P. cirrifera* (12%) *Ektonodiastylis nimia* (10%)	*S. typicus* (73%)	*S. typicus* (49%)
17	37/68	5.4	4.5	0.74	0.09	−0.38	1250	48	*E. nimia* (13%)	*Ctenodiscus crispatus* 49%) *O. borealis* (30%)	*C. crispatus* (16%) *A. malmgreni* (15%)
20	19/33	1.8	2.7	0.54	0.25	−0.43	1870	64	*Y. nana* (42%) *M. ferruginosa* (22%)	*Strongylocentrotus pallidus* (93%)	*Y. nana* (22%)

Note: SR—species richness, α—alpha diversity, H’—Shannon index, J’—Pielou evenness, D’—Simpson index, *D_E_*—difference of evenness index, ES_100_—expected number of species among 100 individuals, *N*—abundance (ind. m^−2^), *B*—biomass (g m^−2^), *R*—respiration rate.

**Table 3:** Frequency of occurrence (FO, %) and abundance (ind. m^−2^)/biomass (g m^−2^) of dominating species with FO > 50% in the Makarov Strait, Barents Sea, autumn 2019.

Taxa	FO	Stations								
		4	5	6	10	11	12	15	17	20
*Yoldiella nana* (Bi)	96	113/0.12	103/0.04	63/0.05	1487/1.71	997/0.72	1393/2.01	27/0.02	313/0.66	780/1.48
*Prionospio cirrifera* (Pol)	96	77/0.09	103/0.09	133/0.20	50/0.06	187/0.36	153/0.35	130/0.23	43/0.06	23/0.04
*Abyssoninoe* cf. *abyssorum* (Pol)	93	57/0.49	23/0.10	13/0.04	67/0.41	177/1.09	67/0.45	80/0.35	37/0.39	30/0.18
*Ektonodiastylis nimia* (Cum)	89	67/0.02	167/0.04	127/0.04	53/0.01	23/0.00	47/0.01	107/0.04	157/0.03	7/0.00
*Mendicula ferruginosa* (Bi)	85	10/0.00	30/0.01	43/0.0.01	1057/0.69	1720/1.24	1043/0.67	0	93/0.06	403/0.34
Cirratulidae g. sp. (Pol)	81	47/0.07	0	13/0.05	60/0.23	100/0.47	30/0.24	10/0.04	50/0.05	17/0.10
*Nephasoma (Nephasoma) diaphanes diaphanes* (Sip)	74	27/0.03	67/0.91	23/0.07	20/0.02	23/0.06	20/0.02	13/0.04	17/0.05	0
*Spiochaetopterus typicus* (Pol)	70	7/1.83	93/13.08	133/12.46	37/1.03	230/14.67	17/0.65	60/12.89	3/0.21	0
*Rabilimis mirabilis* (Ost)	70	10/0.01	13/0.01	7/0.00	20/0.01	30/0.02	183/0.10	17/0.01	7/0.00	30/0.02
*Aglaophamus malmgreni* (Pol)	67	10/0.97	0	3/0.01	33/0.05	0	33/0.13	23/0.49	13/0.96	13/0.07
*Parathyasira equalis* (Bi)	67	13/0.03	7/0.01	10/0.05	297/1.08	663/2.91	347/1.33	0	13/0.06	127/0.77
Nemertini g. sp. (Nem)	67	10/0.22	13/0.11	3/0.02	17/0.05	10/0.05	17/0.01	17/0.08	10/0.01	3/0.00
*Dacrydium vitreum* (Bi)	63	3/0.02	7/0.01	3/0.02	53/0.07	17/0.01	27/	0	10/0.00	20/0.02
*Bathyarca glacialis* (Bi)	56	3/0.05	10/0.06	10/0.08	17/0.21	13/0.23	3/0.00	27/0.64	0	3/0.00
*Yoldiella lenticula* (Bi)	56	37/0.19	20/0.03	0	0	60/0.16	143/0.64	0	3/0.01	270/0.78
*Heteromastus filiformis* (Pol)	56	7/0.01	13/0.13	0	83/0.30	103/0.21	53/0.12	0	13/0.05	13/0.02
*Spiophanes kroeyeri* (Pol)	56	23/0.12	17/0.02	50/0.11	0	3/0.00	0	67/0.30	10/0.01	0
*Pholoe assimilis* (Po)	56	0	47/0/04	17/0.01	0	30/0.02	33/0.02	10/0.01	3/0.00	0
*Yoldiella intermedia* (Bi)	52	0	0	47/0.14	33/0.13	20/0.18	10/0.03	43/0.22	7/1.01	0
*Ophelina cylindricaudata* (Pol)	52	3/0.00	7/0.01	13/0.01	0	7/0.01	0	30/0.02	23/0.02	53/0.04

Note: Bi—Bivalvia, Cum—Cumacea, Nem—Nemertini, Ost—Ostracoda, Pol—Polychaeta, Sip—Sipunculida.

**Table 4:** Benthic community characteristics in the Makarov Strait.

Characteristics	Community				
	Southern Complex		Northern Complex		
	I	II	III	IV	V
SR	96	73	117	82	68
*N*	5110 ±340	3030 ± 590	1300 ± 110	945 ± 110	1250
*B*	55 ± 17	39 ± 18	61 ± 16	11 ± 3	48
H’	3.7 ± 0.2	3.2 ± 0.4	5.2 ± 0.2	4.8 ± 0.1	4.5
J’	0.59 ± 0.1	0.56 ± 0.02	0.83 ± 0.01	0.84 ± 0.01	0.74
D	0.17 ± 0.02	0.21 ± 0.06	0.05 ± 0.00	0.06 ± 0.00	0.09
*D_E_*	0.04 ± 0.03	−0.26 ± 0.16	−0.56 ± 0.10	−0.37 ± 0.14	−0.38
B_inf_:B_epf_	1:1	1:5	1:2.4	4:1	1.5:1
Dominants by respiration rate	*Spiochaetopterus typicus* (18%) *Laonice cirrata* (10%)	*Y. nana* (23%) *Strongylocentrotus pallidus* (18%) *Parathyasira equalis* (10%)	*S. typicus* (46%) *O. borealis* (21%)	*S. typicus* (31%) *Aglaophamus malmgreni* (15%) *Abyssoninoe* cf. *abyssorum* (15%)	*Ctenodiscus crispatus* (17%) *Aglaophamus malmgreni* (13%)
Dominants by biomass	*Ophiopleura borealis* (25%) *S. typicus* (15%)	*S. pallidus* (47%) *O. borealis* (12%) *Y. nana* (8%)	*O. borealis* (40%) *S. typicus* (24%) *Ophiacantha bidentata* (22%)	*S. typicus* (55%) *Aglaophamus malmgreni* (11%)	*Ctenodiscus crispatus* (49%) *O. borealis* (30%)
Dominants by abundance	*Mendicula ferruginosa* (27%) *Yoldiella nana* (24%)	*Y. nana* (36%) *M. ferruginosa* (24%)	*Ektonodiastylis nimia* (11%) *Prionospio cirrifera* (9%)	*Prionospio cirrifera* (11%)	*Y. nana* (25%)
Dominating trophic groups by biomass	Surface deposit feeder (31%) Sub-surface deposit feeder (24%) Carnivores (18%)	Sub-surface deposit feeders (50%)	Sessile suspension feeders (58%)	Sessile suspension feeders (57%)	Mobile suspension feeders (41%) Sub-surface deposit feeders (33%)
Characteristic species	*Maldane sarsi*, *Praxillella gracilis*, *Golfingia (Golfingia) vulgaris vulgaris*, *Y. nana*, *Parathyasira equalis, M. ferruginosa*	*Parathyasira equalis*, *M. ferruginosa*, *Yoldiella lenticula, O. borealis*	*Nephasoma (Nephasoma) diaphanes diaphanes*, *Prionospio cirrifera, Spiophanes kroeyeri*, *Harpinia mucronata*, *Ektonodiastylis nimia*, *Y. nana*, *M. ferruginosa*	*Prionospio cirrifera, Spiophanes kroeyeri, Notomastus latericeus*, *Astarte crenata, Bathyarca glacialis*, *Ektonodiastylis nimia*	*Ophiopleura borealis*, *Y. nana*, *Astarte crenata*,
Depth, m	445–502	392–430	343–507	361–427	186
Stations	10, 11	12, 20	5, 6	4, 15	17

Note: SR—species richness, H’—Shannon index, J’—Pielou evenness, D’—Simpson index, *D_E_*—difference of evenness index, *N*—abundance (ind. m^−2^), *B*—biomass (g m^−2^), B_inf_—infaunal biomass, B_epf_—epifaunal biomass.

**Table 5:** List of environmental variables that contributed to the RDA models based on the benthic abundance and diversity data in the Makarov Strait.

Variable	Abundance			Diversity			
	EV	F	P	Variable	EV	F	P
OM	44	0.001	5.44	OM	36	0.038	4.01
S	7	0.474	0.86	T	11	0.458	0.77
IFP	7	0.499	0.9	Sed	3	0.734	0.25
T	7	0.494	0.82	S	7	0.483	0.62
H	6	0.613	0.57	IFP	3	0.736	0.22
Sed	5	0.738	0.41	H	2	0.806	0.11

Note: T—temperature (°C); S—salinity; OM—organic matter (%); H—depth; Sed—sediments; IFP—duration of ice-free period; EV—explained variation (%); F—pseudo F-ratio; p—probability level.

## Data Availability

The data are available upon request from the corresponding authors.

## Appendix A

**Table A1:** Macrobenthic taxa registered at stations in the Makarov Strait, northeastern Barents Sea. “+” indicates the presence of a taxon.

Taxon	Station								
	4	5	6	10	11	12	15	17	20
**Phylum Porifera**									
**Class Calcarea**									
*Clathrina blanca* (Miklucho-Maclay, 1868)		+					+		
Calcarea g. sp.				+					
**Class Demospongiae**									
*Polymastia grimaldii* (Topsent, 1913)		+				+	+		
*Polymastia* sp.			+			+	+		
Polymastiidae g. sp.			+					+	
**Phylum Cnidaria**									
**Class Hydrozoa**									
*Eudendrium vaginatum* Allman, 1863					+				
*Eudendrium* sp. 1 (*capillare*?)		+							
*Eudendrium* sp. 2 (*album*?)					+				
*Halitholus yoldiaearcticae* (Birula, 1897)						+			
*Ptychogena crocea* Kramp & Damas, 1925		+							
**Class Anthozoa**									
*Gersemia fruticosa* (Sars, 1860)				+	+		+		
*Gersemia rubiformis* (Ehrenberg, 1834)		+		+					
Edwardsiidae g. sp.	+	+					+		
**Phylum Nemertea**									
Nemertea g. sp.	+	+	+	+	+	+	+	+	+
**Phylum Nematoda**									
Nematoda g. sp.	+	+	+	+	+	+	+	+	
**Phylum Annelida**									
**Class Polychaeta**									
*Abyssoninoe* cf. *abyssorum (McIntosh, 1885)*	+	+	+	+	+	+	+	+	+
*Aglaophamus malmgreni* (Théel, 1879)	+		+	+		+	+	+	+
Ampharetidae g. sp.		+					+	+	
*Ampharete finmarchica* (M. Sars, 1866)	+			+					
*Aphelochaeta* sp.	+		+						
*Aricidea hartmanae* (Strelzov, 1968)	+			+		+			+
*Aricidea (Strelzovia) quadrilobata* Webster & Benedict, 1887	+					+	+	+	
*Aricidea nolani* Webster & Benedict, 1887			+						
*Aricidea* sp.			+						
*Artacama proboscidea* Malmgren, 1865								+	
*Bylgides* sp.		+							
*Chone infundibuliformis* Krøyer, 1856		+					+		
*Chone murmanica* Lucash, 1910		+				+	+	+	
*Chone* sp.	+	+							
Cirratulidae g. sp.	+		+	+	+	+	+	+	+
*Cistenides hyperborea* (Malmgren, 1865)				+	+	+			+
*Cossura longocirrata* Webster & Benedict, 1887			+	+	+	+			+
*Dipolydora caeca* (Oersted, 1843)				+					
*Enipo torelli* (Malmgren, 1865)					+				
*Eteone flava* (Fabricius, 1780)		+		+	+	+			
*Euchone analis* (Krøyer, 1856)						+			+
*Galathowenia oculata* Zachs, 1923		+	+	+	+	+			
*Glyphanostomum pallescens* (Théel, 1873)	+	+		+			+		
*Harmothoe impar impar* (Johnston, 1839)		+							
Hesionidae g. sp.			+						
*Heteromastus filiformis* (Claparède, 1864)	+	+		+	+	+		+	+
*Laonice cirrata* (M. Sars, 1851)		+	+	+	+		+	+	
*Laphania boecki* Malmgren, 1865					+				
*Leitoscoloplos acutus* (Verrill, 1873)				+	+	+			+
*Levinsenia gracilis* (Tauber, 1879)				+	+	+			
*Lysippe labiata* Malmgren, 1865				+	+				
*Maldane sarsi* Malmgren, 1867	+		+	+	+				
Maldanidae g. sp.			+						
*Melinna elisabethae* McIntosh, 1922								+	
*Melinnopsis arctica* (Annenkova, 1931)							+		
*Myriochele heeri* Malmgren, 1867		+	+		+	+	+	+	
*Nephtys ciliata* (Müller, 1779)		+		+	+				
*Nephtyidae* g. sp.		+		+					
*Nereis zonata* Malmgren, 1867		+			+				
*Nothria hyperborea* (Hansen, 1878)		+		+				+	
*Notomastus latericeus* M. Sars, 1851	+	+	+				+		
*Notoproctus oculatus* Arwidsson, 1906		+	+			+		+	
*Ophelina abranchiata* Støp-Bowitz, 1948	+	+			+	+		+	
*Ophelina cylindricaudata* (Hansen, 1878)	+	+	+		+		+	+	+
Owenidae g. sp.		+	+				+	+	
*Parougia* sp.	+	+	+	+			+	+	
*Pholoe assimilis* (Muller, 1776)		+	+	+	+	+	+	+	
*Phyllodoce groenlandica* Oersted, 1842			+		+		+		
*Phyllodocidae* g. sp.				+					
*Polycirrus medusa* Grube, 1850						+			
Polynoidae g. sp.		+	+						
*Praxillella gracilis* (M. Sars, 1861)			+	+	+	+		+	
*Praxillella praetermissa* (Malmgren, 1865)		+						+	+
*Praxillura longissima* Arwidsson, 1906		+			+		+	+	
*Prionospio cirrifera* (Wirén, 1883)	+	+	+	+	+	+	+	+	+
*Pseudoscalibregma parvum* (Hansen, 1878)	+		+						
*Scalibregma inflatum* Rathke, 1843		+		+	+	+		+	+
*Scolelepis aggr. korsuni* Sikorski, 1994		+							
*Sosane wireni* (Hessle, 1917)	+	+					+		
*Sphaerodoropsis philippi* (Fauvel, 1911)	+		+			+	+	+	
*Sphaerodoridium kolchaki* Gagaev, 2015				+	+				
*Spio limicola* Verrill, 1879				+	+				
*Spiochaetopterus typicus* M. Sars, 1856	+	+	+	+	+	+	+	+	
*Spiophanes kroeyeri* Grube, 1860	+	+	+		+		+	+	
Syllidae g. sp.							+	+	
*Syllis cornuta* Rathke, 1843								+	
*Thelepus cincinnatus* (Fabricius, 1780)		+							
Terebellidae g. sp.		+					+		
*Terebellides stroemi* Sars, 1835	+	+	+		+		+	+	
*Terebellides gracilis* Malm, 1874							+		
*Trochochaeta carica* Birula, 1879			+						
**Subclass Echiura**									
*Hamingia arctica* Danielsen & Koren, 1881	+								
**Phylum Sipuncula**									
**Class Sipunculidea**									
*Golfingia (Golfingia) vulgaris vulgaris* (de Blainville, 1827)				+	+	+			+
*Golfingia (Golfingia)* sp.				+					
*Nephasoma (Nephasoma) capilleforme* (Murina, 1973)								+	
*Nephasoma (Nephasoma) diaphanes diaphanes* (Gerould, 1913)	+	+	+	+	+	+	+	+	
*Nephasoma (Nephasoma) lilljeborgi* (Danielssen & Koren, 1880)	+	+	+		+				
*Nephasoma (Nephasoma)* sp.	+		+						
*Phascolion (Phascolion) strombus strombus* (Montagu, 1804)				+	+	+			
**Phylum Arthropoda**									
**Class Malacostraca**									
**Order Amphipoda**									
*Aceroides (Aceroides) latipes* (G.O. Sars, 1882)							+		
*Arrhis phyllonyx* (M. Sars, 1858)		+	+				+		
*Centromedon calcaratus* G. O. Sars, 1879			+						
*Centromedon typhlops* (G. O. Sars, 1879)					+	+			
*Centromedon* sp.			+						
*Halice abyssi* Stappers, 1911			+						
*Haploops tenuis* Kanneworff, 1966			+		+	+			
*Haploops* sp.		+							
*Harpinia mucronata* G.O. Sars, 1879		+	+			+	+	+	
*Harpinia pectinata* G.O. Sars, 1891		+	+						
*Harpiniopsis similis* Stephensen, 1925				+					
*Hippomedon* sp.						+			
Lysianassidae g. sp.	+								
*Paraphoxus oculatus* (G.O. Sars, 1879)				+					
*Pardaliscella boecki* (Malm, 1870)								+	
*Paroediceros lynceus* (M. Sars, 1858)	+								
*Pleusymtes pulchella* (G.O. Sars, 1876)				+					
*Pontoporeia femorata* Krøyer, 1842					+				
**Order Cumacea**									
*Ektonodiastylis nimia* (Hansen, 1920)	+	+	+	+	+	+	+	+	+
*Eudorella emarginata* (Krøyer, 1846)				+	+	+			+
*Eudorella gracilis* Sars, 1871	+	+	+				+		
*Eudorella* sp.								+	
*Leptostylis gorbunovi* Zimmer, 1946			+						
*Leucon (Leucon) acutirostris* G.O. Sars, 1864			+		+	+		+	+
*Leucon (Leucon) nasica* (Krøyer, 1841)					+				
*Leucon (Alytoleucon) pallidus* G.O. Sars, 1864	+					+		+	
**Order Decapoda**									
*Chionoecetes opilio* juv. (O. Fabricius, 1788)					+				
**Order Isopoda**									
*Caecognathia elongata* (Krøyer, 1847)		+						+	
*Calathura brachiata* (Stimpson, 1854)		+						+	
Desmosomatidae g. sp.							+	+	
*Ilyarachna bicornis* Hansen, 1916			+						
Ilyarachninae g. sp.			+					+	
**Order Tanaidacea**									
*Akanthophoreus* sp.				+					
*Chauliopleona armata* (Hansen, 1913)			+				+		
*Pseudotanais affinis* Hansen, 1887			+						
*Pseudotanais lilljeborgii* Sars, 1882				+	+				
*Pseudosphyrapus anomalus* (Sars, 1869)	+	+	+			+	+	+	
*Pseudotanais* sp.		+							
*Typhlotanais* sp.			+						
**Class Ostracoda**									
*Heterocyprideis sorbyana* (Jones, 1857)				+	+		+		
*Philomedes globosus* (Lilljeborg, 1853)				+	+	+			
*Rabilimis mirabilis* (Brady,1968) Hazel, 1967	+	+	+	+	+	+	+	+	+
**Class Pycnogonida**									
*Nymphon hirtipes* Bell, 1855			+						
**Phylum Mollusca**									
**Class Bivalvia**									
*Astarte crenata* (Gray, 1842)	+	+	+	+		+	+	+	
*Axinopsida orbuculata* (G.O. Sars, 1878)						+		+	
*Bathyarca glacialis* (Gray, 1842)	+	+	+	+	+	+	+		+
*Ciliatocardium ciliatum* (Fabricius, 1780)		+		+	+	+			
*Cuspidaria glacialis* (G.O. Sars, 1878)		+	+		+	+	+	+	
*Dacrydium vitreum* (Møller, 1842)	+	+	+	+	+	+		+	+
*Ennucula tenuis* (Montagu, 1808)				+	+				+
*Macoma calcarea* (Gmelin,1791)				+	+	+			+
*Mendicula ferruginosa* (Forbes, 1844)	+	+	+	+	+	+		+	+
*Nuculana pernula* (O.F. Müller, 1779)				+	+	+			+
*Parathyasira equalis* (Verrill & Bush, 1898)	+	+	+	+	+	+		+	+
*Yoldiella annenkovae* (Gorbunov, 1946)	+	+	+			+	+		
*Yoldiella frigida* (Torell, 1859)				+	+	+			+
*Yoldiella intermedia* (M. Sars, 1865)			+	+	+	+	+	+	
*Yoldiella lenticula* (Møller, 1842)	+	+			+	+		+	+
*Yoldiella nana* (M. Sars, 1865)	+	+	+	+	+	+	+	+	+
*Yoldiella solidula* Warén, 1989						+			
**Class Caudofoveata**									
Caudofoveata g. sp.				+	+	+			
**Class Gastropoda**									
*Cylichna alba* (T. Brown, 1827)			+	+				+	
*Diaphana hiemalis* (Couthouy, 1839)						+		+	
*Frigidoalvania janmayeni* (Friele, 1878)						+			
*Lepeta coeca* (O.F. Müller, 1776)									
*Margarites groenlandicus* (Gmelin, 1791)									
*Onoba aculeus* (A. Gould, 1841)		+							
*Onoba leptalea* (Verrill, 1884)		+							
*Propebela harpularia* (Couthuoy, 1838)		+	+	+	+	+	+	+	+
*Punctulum wyvillethomsoni* (Friele, 1877)		+			+	+	+	+	
*Retusa obtusa* (Montagu, 1803)	+					+			
*Testudinalia testudinalis* (O.F. Müller, 1776)					+				
**Class Scaphopoda**									
*Siphonodentalium lobatum* (G.B. Sowerby II, 1860)	+								
**Phylum Bryozoa**									
**Class Gymnolaemata**									
*Alcyonidium gelatinosum* (Linneus, 1761)							+		
*Alcyonidium radicellatum* Kluge, 1946		+					+		
*Bugulopsis peachii* (Busk, 1851)							+		
*Copidozoum smitti* (Kluge, 1946)		+					+		
*Escharoides jacksonii* (Waters, 1900)								+	
*Eucratea loricata* (Linneus, 1758)		+					+	+	
*Leieschara subgracilis* (d’Orbigny, 1853)		+							
*Parasmittina jeffreysii* (Norman, 1876)					+				
*Pseudoflustra solida* (Stimpson, 1854)							+		
*Sarsiflustra abyssicola* (G.O. Sars, 1872)		+							
*Schizoporella costata* Kluge, 1962				+					
*Scrupocellaria intermedia* Norman, 1893								+	
*Scrupocellaria minor* Kluge, 1915		+							
*Stomacrustula cruenta* (Busk, 1854)									
*Uschakovia gorbunovi* Kluge, 1946		+							
**Class Stenolaemata**									
*Crisia eburneodenticulata* Smitt ms in Busk, 1875					+	+		+	
*Crisiella* sp.		+							
*Exidmonea atlantica* (Forbs in Jonston, 1847)		+							
**Phylum Echinodermata**									
**Class Asteroidea**									
*Ctenodiscus crispatus* (Bruzelius, 1805)				+	+			+	+
*Pontaster tenuispinus* (Düben & Koren, 1846)		+							
**Class Echinoidea**									
*Strongylocentrotus pallidus* (G.O. Sars, 1872)									+
**Class Holothuroidea**									
*Chiridota laevis* (Fabricius, 1780)		+							
*Molpadia arctica* (Marenzeller von, 1877)				+					
*Myriotrochus eurycyclus* Heding, 1935		+							
*Myriotrochus rinkii* Steenstrup, 1851								+	
**Class Ophiuroidea**									
*Gorgonocephalus arcticus* Leach, 1819				+	+				
*Ophiacantha bidentata* (Bruzelius, 1805)		+			+			+	
*Ophiopus arcticus* Ljungman, 1867					+				
*Ophiocten sericeum* (Forbes, 1852)								+	
*Ophiopleura borealis* Danielssen & Koren, 1877			+	+	+	+		+	
*Ophioscolex glacialis* Müller & Troschel, 1842		+	+				+		
Ophiuroidea g. sp.		+				+			
**Phylum Chordata**									
**Class Ascidiacea**									
*Molgula* sp.					+				
*Polycarpa comata* (Alder, 1863)						+			
